# Analysis of myosin genes in HNSCC and identify MYL1 as a specific poor prognostic biomarker, promotes tumor metastasis and correlates with tumor immune infiltration in HNSCC

**DOI:** 10.1186/s12885-023-11349-5

**Published:** 2023-09-07

**Authors:** Ce Li, Rui Guan, Wenming Li, Dongmin Wei, Shengda Cao, Fen Chang, Qun Wei, Ran Wei, Long Chen, Chenyang Xu, Kainan Wu, Dapeng Lei

**Affiliations:** Department of Otorhinolaryngology, Qilu Hospital, NHC Key Laboratory of Otorhinolaryngology (Shandong University), Shandong University, 107 West Wenhua Road, Jinan, 250012 Shandong China

**Keywords:** HNSCC, Prognostic marker, MYL1, Myosin genes, Metastasis

## Abstract

Head neck squamous cell carcinoma (HNSCC) is one of the most common malignant tumors which ranks the sixth incidence in the world. Although treatments for HNSCC have improved significantly in recent years, its recurrence rate and mortality rate remain high. Myosin genes have been studied in a variety of tumors, however its role in HNSCC has not been elucidated. GSE58911 and GSE30784 gene expression profile analysis were performed to detect significantly dys-regulated myosin genes in HNSCC. The Cancer Genome Atlas (TCGA) HNSCC database was used to verify the dys-regulated myosin genes and study the relationship between these genes and prognosis in HNSCC. The results showed that MYL1, MYL2, MYL3, MYH2, and MYH7 were down-regulated, while MYH10 was up-regulated in patients with HNSCC. Interestingly, MYL1, MYL2, MYH1, MYH2, and MYH7 were shown to be unfavorable prognostic markers in HNSCC. It is also worth noting that MYL1 was a specific unfavorable prognostic biomarker in HNSCC. MYL1, MYL2, MYL3, MYH2, MYH7, and MYH10 promoted CD4 + T cells activation in HNSCC. MYL1 was proved to be down-regulated in HNSCC tissues compared to normal tissues at protein levels. MYL1 overexpression had no effect on proliferation, but significantly promoted migration of Fadu cells. MYL1 increased EGF and EGFR protein expression levels. Moreover, there is a positive correlation between MYL1 expression and Tcm CD8 cells, Tcm CD4 + cells, NK cells, Mast cells, NKT cells, Tfh cells and Treg cells in HNSCC. Overall, MYL1 facilitates tumor metastasis and correlates with tumor immune infiltration in HNSCC and these effects may be associated with the EGF/EGFR pathway.

**Supplementary Information:**

The online version contains supplementary material available at 10.1186/s12885-023-11349-5.

## Introduction

Squamous cell carcinoma of the head and neck (HNSCC) is the sixth most common malignant tumor in the world and includes squamous cell carcinoma of the lips and mouth, nasal cavity, sinuses, oropharynx, larynx and nasopharynx, with nearly 700,000 new cases and 380,000 deaths worldwide each year [1]. For early HNSCC, surgery and radiotherapy are the main necessary treatments. But 70 to 80% of patients are already at locally advanced stage or end-stage, and for such patients, treatment is through surgery or chemoradiotherapy. After treatment, the 5-year survival rate is only 40% or lower [2]. Therefore, it is important to search for novel tumor therapeutic targets and prognostic markers.

Myofibrillar proteins are key components of the myofibrils that make up muscles. ​It consists mainly of tropomyosin, myosin, myogenin, actomyosin, and other types. Human myosin, which contains 2 myosin heavy chains and 4 myosin light chains, is an essential component of muscle tissue. It can convert chemical energy into mechanical energy by hydrolyzing ATP, and cooperates with actin to induce the contraction of myofibrils [3]. Human myosin light chain is regulated by the MYL gene family, and human myosin heavy chain is regulated by the MYH gene family [5]. Most current research on the relationship between myosin genes and tumors remains at the bioinformatics level, lacking systematic experimental and mechanistic validation.  MYL1 was identified as a significant gene markers for rhabdomyosarcoma and may be involved in the pathogenesis of rhabdomyosarcoma [6]. Shaikh et al. found that muscle contraction was the most abundant biological process in both smoking (TOB) and non-smoking (N-TOB) HNSCC samples, with associated genes including MYL2 [7]. Deng et al. found that MYL3 was a candidate prognostic biomarkers for Ewing’s sarcoma [8]. Zhang et al. found that MYL5 expression level was up-regulated in patients with advanced cervical cancer, positively correlated with pelvic lymph node metastasis, and was identified as a poor survival indicator [9]. MYL9 was identified as a fibroblast-specific biomarker of poor prognosis in colorectal cancer [10]. MYH2 was proved as a marker in distinguishing head and neck squamous cell carcinoma and lung squamous cell carcinoma [11]. MYH8 truncation may be a novel prognostic marker associated with poor prognosis [12]. MYH9 plays a dual role in tumors. MYH9 was closely associated with the progression and poor prognosis of most solid tumors, but proved as a suppressor gene via regulating the re-Rho pathway [13]. MYH10 silencing was proved to reduce cell migration and invasion in the glioma cells via inhibiting the Wnt/β-Catenin pathway [14]. MYH9 and MYH14 were highly expressed, while MYH10 was under-expressed in pancreatic cancer compared to adjacent normal tissues. MYH14 promoted metastasis in pancreatic cancer [15]. However, the role of the myosin gene in HNSCC has not been elucidated and needs further study.

This study was performed using gene expression profiles and the TCGA database. The GSE58911 and GSE30784 databases were used to detect significantly dysregulated myosin genes in the HNSCC and the TCGA HNSCC database was used to validate these myosin genes. Gene methylation analysis and survival correlation analysis were also performed by using the TCGA HNSCC database. Myosin genes MYL1, MYL2, MYH2, and MYH7 were proved significantly down-regulated but as unfavorable prognostic markers and related with tumor stages or grades in HNSCC. MYL1 promoted HSCC metastasis and correlated with tumor immune infiltration in HNSCC, these functions may be related to the EGF/EGFR signaling pathway. These results may contribute to further understanding of the role of myosin genes in the occurrence and development of HNSCC and provide a basis for exploring its diagnostic and therapeutic targets.

## Materials and methods

### Chip detection and bioinformatics screening

Gene expression profiles (GSE58911 and GSE30784) analysis was performed to detect significantly up-regulated and down-regulated genes in HNSCC by using R studio 4.1.1 and limma package. Volcanic map of genetic differences was performed by using pheatmap package.

### Gene expression analysis in HNSCC patients and normal persons

Gene expression analysis of HNSCC patients in the Cancer Genome Atlas (TCGA) database was performed by using GEPIA. (http://gepia.cancer-pku.cn/) [16]. GEPIA (Gene Expression Profiling Interactive Analysis) is an interactive web server for analyzing the RNA sequencing expression data of 9,736 tumors and 8,587 normal samples from the TCGA and the GTEx projects, using a standard processing pipeline.

### Gene methylation level analysis and protein expression analysis in HNSCC patients and normal persons

Gene methylation level of HNSCC patients in TCGA database was performed by using UALCAN (http://ualcan.path.uab.edu/index.html) [17]. UALCAN is built on PERL-CGI for high quality graphics using JavaScript and CSS and used to analyze cancer-omics data. UALCAN is designed to provide easy access to publicly available cancer-omics data (TCGA, MET500, CPTAC, and CBTTC), and allow users to identify biomarkers or perform computer validation of potentially interesting genes. Protein expression analysis in HNSCC patients and normal persons was performed by using UALCAN proteomics analysis (https://ualcan.path.uab.edu/analysis-prot.html). UALCAN provides protein expression analysis option using data from Clinical Proteomic Tumor Analysis Consortium (CPTAC) and the International Cancer Proteogenome Consortium (ICPC) datasets.

### Prognostic survival analysis and correlation analysis between gene expression and tumor stage or grade

Correlation analysis between gene expression and HNSCC patients overall survival time as well as disease-free survival time in TCGA database were performed by using GEPIA (http://gepia.cancer-pku.cn/) [16]. Correlation analysis between gene expression and HNSCC tumor stage in TCGA database was performed by using GEPIA (http://gepia.cancer-pku.cn/) [16]. Correlation analysis between gene expression and HNSCC tumor grade in TCGA database was performed by using UALCAN [17]. Correlation analysis between expression levels of myosin genes and overall survival time of different types of tumors were performed by using GEPIA2 (http://gepia2.cancer-pku.cn/) [18]. GEPIA 2 (Gene Expression Profiling Interactive Analysis 2) is an online gene expression analysis platform. The primary mission of GEPIA 2 is to collect, store, manage and distribute gene expression data from a variety of organisms to support and service life science research.

### Functional protein association networks and anaysis of MYL1 relevant genes

Functional protein association networks were performed by using

GeneMANIA (http://genemania.org/) [19]. GeneMANIA finds other genes that are related to a set of input genes, using a very large set of functional association data. Association data include protein and genetic interactions, pathways, co-expression, co-localization and protein domain similarity.

Correlation analysis of MYL1 and relevant genes in TCGA HNSCC database by using GEPIA (http://gepia.cancer-pku.cn/) [16].

### Tumor-infiltrating lymphocytes (TILs) analysis

Correlations between prognostic gene expression levels and immune infiltrations in HNSCC were performed by using TIMER (https://cistrome.shinyapps.io/timer/) [20, 21]. TIMER web server is a comprehensive resource for systematical analysis of immune infiltrates across diverse cancer types. The abundances of six immune infiltrates (B cells, CD4 + T cells, CD8 + T cells, Neutrophils, Macrophages, and Dendritic cells) were estimated by TIMER algorithm. TIMER web server allows users to input function-specific parameters, with resulting figures dynamically displayed to conveniently access the tumor immunological, clinical, and genomic features. Correlations between prognostic MYL1 expression levels and Tumor-infiltrating lymphocytes (TILs) in HNSCC were performed by using TISIDB (http://cis.hku.hk/TISIDB/) [22]. TISIDB is a web portal for tumor and immune system interaction, which integrates multiple heterogeneous data types.

### Cell lines, cell culture and tissues

Fadu, TU686, TU212 and Detroit562 cells were obtained from American Type Culture Collection. Cells were cultured in DMEM high glucose (BasalMedia, Shanghai, China) containing 10% fetal bovine serum (Sigma, Saint Louis, USA). Hypopharygeal squamous cell carcinoma (HSCC) tissues and adjacent normal tissues were obtained from department of Otorhinolaryngology of Qilu Hospital. All patient material obtained for this study was approved by the Ethics Committee of Qilu Hospital of Shandong University.

### Antibodies and plasmids

The following antibodies were used for western blot: MYL1 (Abcam, ab151749, 1:1000), EGF (Abcam, ab206423, 1:1000), EGFR (Abcam, EP38Y, 1:1000), β-actin (Abcam, ab8226, 1:1000), Goat anti-mouse second antibody (Cell Signaling Technology, 5470 S, 1:15000), Goat anti-rabbit second antibody (Cell Signaling Technology, 5151 S, 1:30000). PENTER control plasmid (Shandong Vigene Biosciences, Jinan, China) and pENTER-MYL1 plasmid (Shandong Vigene Biosciences, Jinan, China) were used for transfection.

### Western blot

Whole cell lysate or tissue was prepared by using RIPA lysis buffer (Beyotime Biotechnology, Shanghai, China) containing a protease inhibitor. The protein was isolated by gel electrophoresis and transferred to PVDF membrane (Millipore, Billerica, USA), then put it in 5% BSA (Sigma, Saint Louis, USA) for 1 h. The membrane was placed in a closed buffer containing primary antibody overnight at 4 °C. Washed and incubated in TBST (Beyotime Biotechnology, Shanghai, China) containing fluorescent secondary antibodies at room temperature for 1 h. After washing, an infrared fluorescence scanning imager was used to detect proteins.

### CCK8 assay

Cell proliferation ability was measured by using CCK8 assay (Beyotime Biotechnology, Shanghai, China) .3 × 10^3^ cells were inoculated in 96-well plates and cultured for 0, 24, 48, 72, and 96 h, respectively. 10µL CCK-8 solution was added to each well and incubated at 37 °C for 1 h, then measure the absorbance at 450 nm.

### Colon assay

Digest cells at logarithmic growth phase and inoculated 300 cells into 6 well plates, cultured in DMEM medium with 10%FBS for 2 weeks; inhaled supernatant and washed with PBS for 3 times; methanol fixed 30 min, stained by using crystal violet for 30 min. More than 30 cells were recorded as clones, counted under microscope and statistically analyzed.

### Wound healing assay

The cells were cultured in 6-well plates. When they reached 100% confluence, the scratches were formed by using 200µL fluid suction heads on the petri dish and cells were cultured in serum-free DMEM medium for 24 h. Cell images were taken 0, 24 and 48 h after the scratch.

### Transwell migration assay

Transwell cell migration kit (Corning, New York, USA) was used for migration detection. 3*10^4^ cells were inoculated into the upper compartment with serum-free medium. It was cultured in the lower chamber with 20% FBS. After 48 h, the cells on the lower surface were fixed with methanol for 30 min and stained with crystal violet for 30 min, then removed the upper membrane, The cells adhering to the lower surface were washed with PBS. Images of the stained cells were taken using a microscope.

### Transcriptome sequencing and analysis

Transcriptome sequencing and analysis were performed by using HiSeq3000 (Illumina, San Diego, USA). The transcriptome sequencing was as follows: total RNA extraction and mRNA separation, library building reagent and quantification, library recovery and bridge amplification were performed by using NanoPhotometer NP80 (Thermo Fisher Scientific, MA, USA), library recovery and bridge amplification were performed by using RNA 6000 Nano Assay Kit (Agilent Technologies, CA, USA) and Bio-RAD KIT iQ SYBR GRN (Bio-Rad, California, USA), then performed sequencing by using computer; the analysis process was as follows: data output and transcriptome splicing, SSR analysis and SNP analysis, gene function annotation and gene expression differential analysis, differential gene expression pattern clustering and differential gene enrichment analysis.

### Statistical analysis

The sample size in each experiment is sufficient to ensure the validity of the statistical results. At least three results of independent experiments were quantified by an uninformed observer and expressed as mean ± SD (standard deviation). SPSS 22.0 statistical software package was used for statistical analysis. Statistical differences between groups were assessed using a two-tailed Student’s t-test. Data conformed to normal distribution and P < 0.05 was considered statistically significant.

## Results

### Analysis of chip-screened dys-regulated myosin genes expression and their correlation with survival time in TCGA HNSCC database

**Fig. 1:**
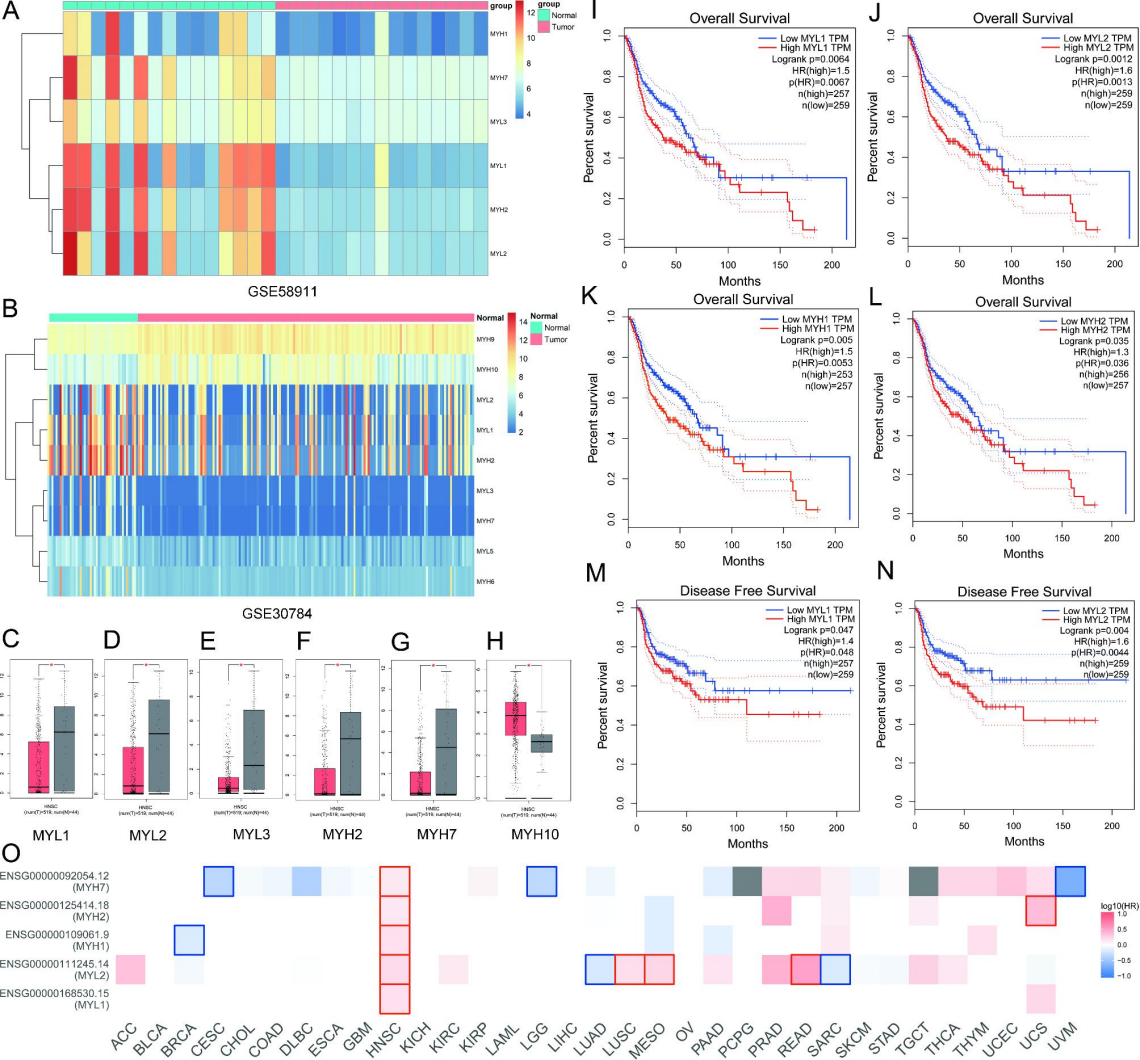
Gene chip analysis of myosin genes in head and neck squamous cell carcinoma and expression level and survival time analysis in TCGA database. **A** Heatmap and hierarchical clustering analysis revealed different myosin genes between 15 pairs of HNSCC tissues and adjacent normal tissues in GSE58911 (log_2_FC > 1.0, P < 0.05). **B** Heatmap and hierarchical clustering analysis revealed different myosin genes between 167 oral squamous cell carcinoma (OSCC) patients and 45 normal tissues in GSE30784 (log_2_FC > 1.0, P < 0.05). **C** MYL1 expression level (log_2_(TPM + 1)) in HNSCC patients and normal persons in TCGA HNSCC database. **D** MYL2 expression level (log_2_(TPM + 1)) in HNSCC patients and normal persons in TCGA HNSCC database. **E** MYL3 expression level (log_2_(TPM + 1)) in HNSCC patients and normal persons in TCGA HNSCC database. **F** MYH2 expression level (log_2_(TPM + 1)) in HNSCC patients and normal persons in TCGA HNSCC database. **G** MYH7 expression level (log_2_(TPM + 1)) in HNSCC patients and normal persons in TCGA HNSCC database. **H** MYH10 expression level (log_2_(TPM + 1)) in HNSCC patients and normal persons in TCGA HNSCC database. **I** Correlation analysis of survival and MYL1 expression in HNSCC patients in TCGA HNSCC database. **J** Correlation analysis of survival and MYL2 expression in HNSCC patients in TCGA HNSCC database. **K** Correlation analysis of survival and MYH1 expression in HNSCC patients in TCGA HNSCC database. **L** Correlation analysis of survival and MYH2 expression in HNSCC patients in TCGA HNSCC database. **M** Correlation analysis of disease free survival and MYL1 expression in HNSCC patients in TCGA by using GEPIA. **N** Correlation analysis of disease free survival and MYL2 expression in HNSCC patients in TCGA by using GEPIA. **O** Overall survival analysis of dys-regulated myosin genes in different types of tumor patients

Gene expression profiles GSE 58,911, which including 15 pairs of head and neck squamous cell carcinoma (HNSCC) tissues and the corresponding tissues adjacent cancers, as well as GSE30784, which including 167 oral squamous cell carcinoma (OSCC) tissues and 45 normal tissues, were used to analyze differentially expressed myosin genes between HNSCC patients and normal persons. The results showed myosin genes MYL1, MYL2, MYL3, MYH1, MYH2, and MYH7 were significantly down-regulated in GSE58911 (Fig. 1A); myosin genes MYL1, MYL2, MYL5, MYH2, MYH6, and MYH7 were significantly down-regulated in GSE30784, while MYH9 and MYH10 were significantly up-regulated in GSE30784 (Fig. 1B). We studied expression levels of these myosin genes in HNSCC patients by using TCGA HNSCC database. The results showed MYL1, MYL2, MYL3, MYH2, and MYH7 were significantly down-regulated (Fig. 1C-G), while MYH10 was significantly up-regulated in TCGA HNSCC database (Fig. 1H). MYL5, MYH1, MYH6, and MYH9 showed no significant changes in TCGA HNSCC database (Figure S1A-D). We then studied overall survival time and disease-free survival time of dys-regulated myosin genes in HNSCC TCGA database. The results showed high expression levels of MYL1, MYL2, MYH1, and MYH2 were related to poor prognosis and shorter overall survival time (p < 0.05) (Fig. 1I-L), MYH7 may be associated with prognosis (HR = 1.3), but showed no significant difference (P = 0.054) (Figure S1E), while MYL3, MYL5, MYH6, MYH9, and MYH10 were not associated with overall survival (p > 0.05) (Figure S1F-J). High MYL1 and MYL2 expression levels were related to shorter disease-free time (p < 0.05) (Fig. 1M, N). Moreover, MYL1 was a potential special poor prognostic marker in HNSCC (Fig. 1O).

### Correlation analysis of tumor stage or grade with dysregulated myosin genes in HNSCC patients

**Fig. 2:**
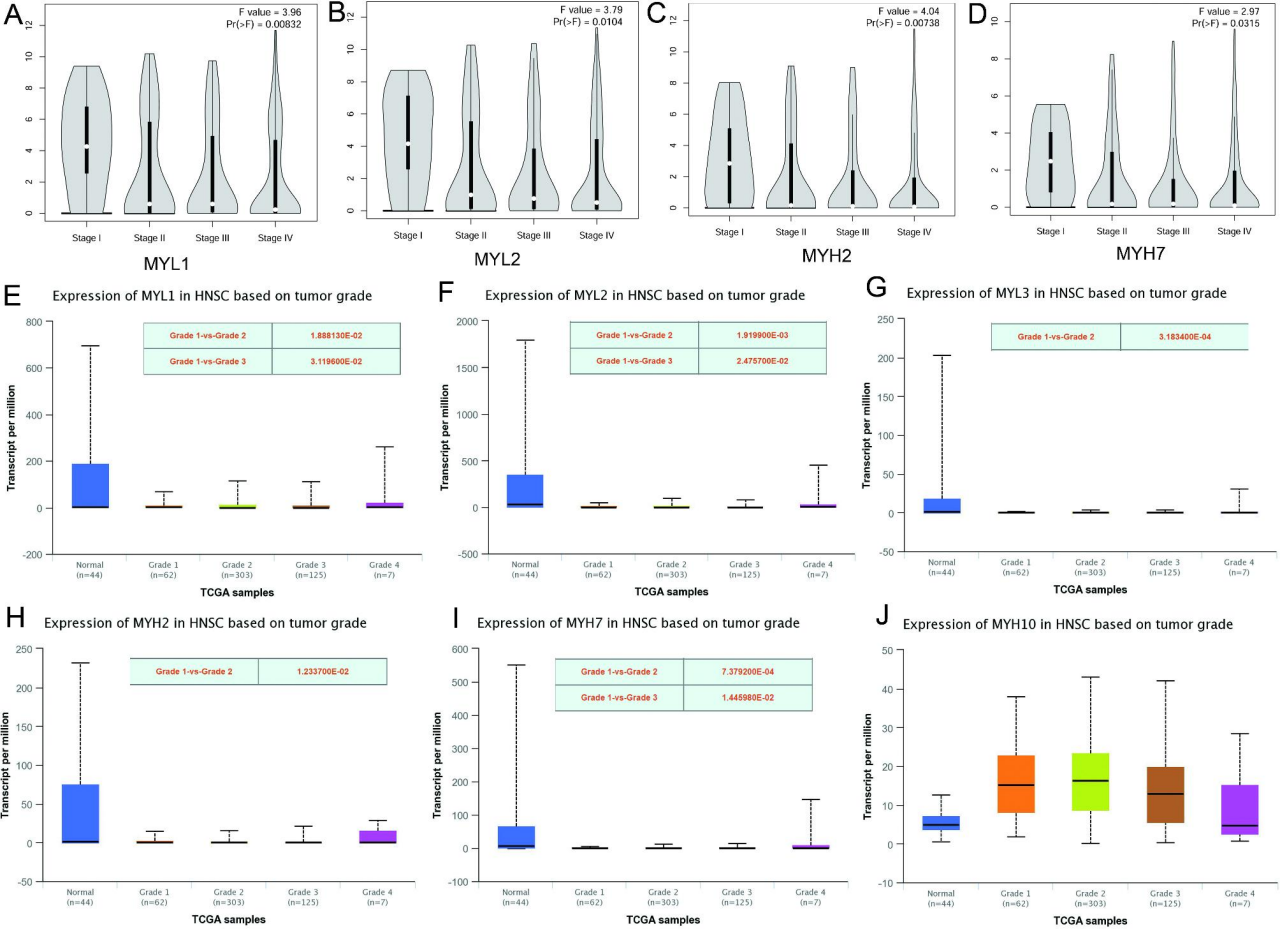
Correlation analysis between tumor stages or grades and expression levels of dys-regulated myosin genes. **A** Correlation analysis of MYL1 and tumor stages in TCGA HNSCC database. **B** Correlation analysis of MYL2 and tumor stages in TCGA HNSCC database. **C** Correlation analysis of MYH2 and tumor stages in TCGA HNSCC database. **D** Correlation analysis of MYH7 and tumor stages in TCGA HNSCC database. **E** Correlation analysis of MYL1 and tumor grades in TCGA HNSCC database. **F** Correlation analysis of MYL2 and tumor grades in TCGA HNSCC database. **G** Correlation analysis of MYL3 and tumor grades in TCGA HNSCC database. **H** Correlation analysis of MYH2 and tumor grades in TCGA HNSCC database. **I** Correlation analysis of MYH7 and tumor grades in TCGA HNSCC database. **J** Correlation analysis of MYH10 and tumor grades in TCGA HNSCC database

We then focus on the dys-regulated genes in HNSCC TCGA database for further study, including MYL1, MYL2, MYL3, MYH2, MYH7, and MYH10. We studied the relationships between dys-regulated myosin genes and tumor stages in the TCGA HNSCC database. The results showed the expression levels of MYL1, MYL2, MYH2, and MYH7 were significantly down-regulated in stage II, III, IV compared with stage I (Fig. 2A-D). However, there are no significant differences between MYL3 or MYH10 and tumor stages (Figure S2A, B). We also studied the relationships between dys-regulated myosin genes and tumor grades in the TCGA HNSCC database (Fig. 2E-J). The results showed the expression levels of MYL1, MYL2, MYL3, MYH2, and MYH7 were significant down-regulated in grade 2 compared with grade 1 (Fig. 2E-I), the expression levels of MYL1, MYL2, and MYH7 were significant down-regulated in grade 3 compared with grade 1 (Fig. 2E, F, I). However, there are no significant differences between grades in MYH10 (Fig. 2J).

### Immune infiltration analysis and methylation level analysis of dys-regulated myosin genes in HNSCC

**Fig. 3:**
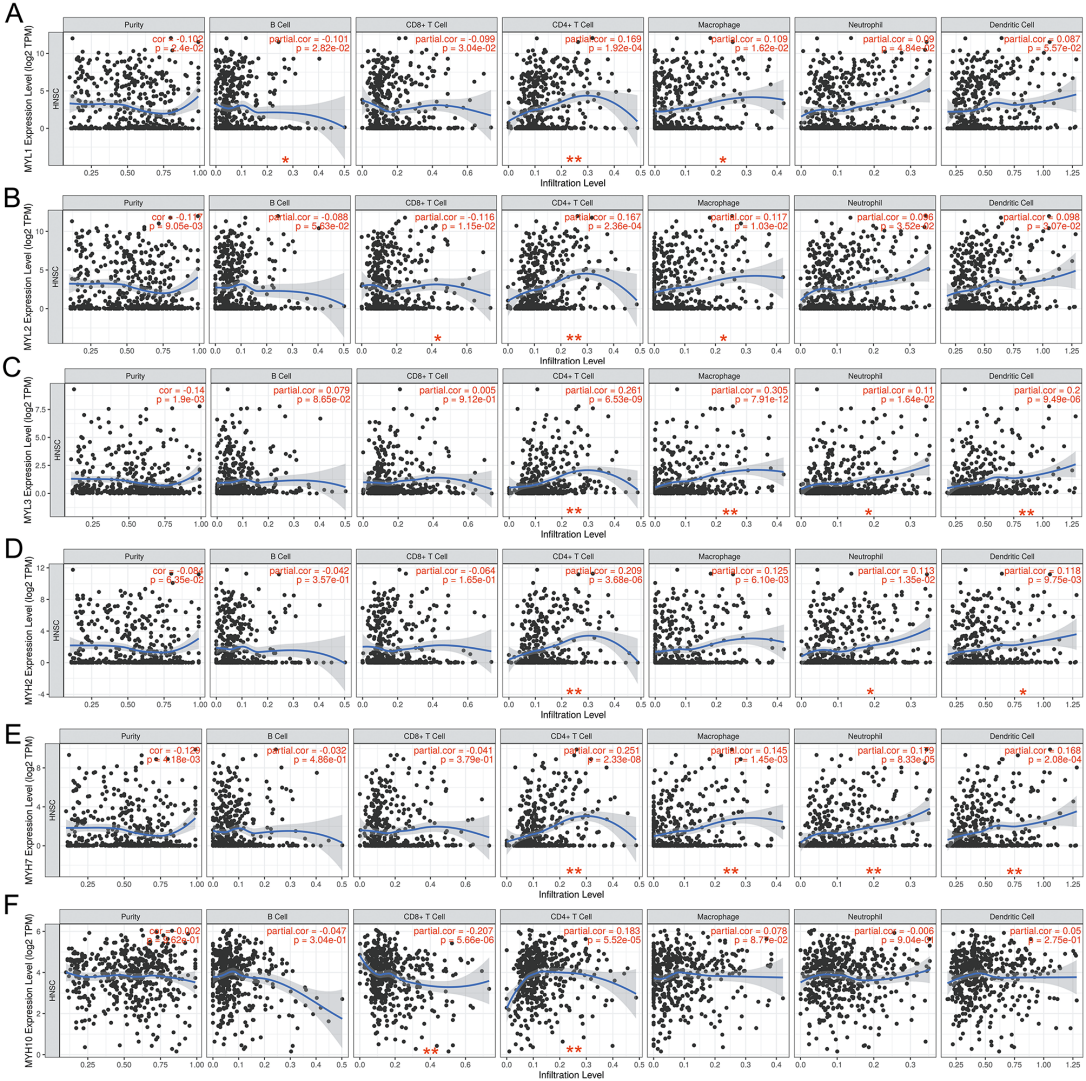
Immune infiltration of myosin genes in HNSCC. **A** Immune infiltration of MYL1 in HNSCC. **B** Immune infiltration of MYL2 in HNSCC. **C** Immune infiltration of MYL3 in HNSCC. **D** Immune infiltration of MYH2 in HNSCC. **E** Immune infiltration of MYH7 in HNSCC. **F** Immune infiltration of MYH10 in HNSCC. (*: P < 0.05, **: p < 0.01)

We then studied immune infiltration of myosin genes in HNSCC. There is a positive relationship between MYL1, MYL2, MYL3, MYH2, MYH7, MYH10 expression levels and infiltration level of CD4 + T cells (r>0.1, P<0.01) (Fig. 3A-F). There is a positive relationship between MYL3, MYH7 expression levels and infiltration level of macrophage cells (r>0.1, P<0.01) (Figure 3C, E). There is a positive relationship between MYL3, MYH7 expression levels and infiltration level of dendritic cells (r>0.1, P<0.01) (Figure 3C, E).  There is a positive relationship between MYH7 expression levels and infiltration level of neutrophil cells (r>0.1, P<0.01) (Fig. 3E). In addition, there is a negative relationship between MYH10 expression level and infiltration level of CD8 + T cells (r<-0.1, P<0.01) (Fig. 3F). These results indicated myosin genes were involved in immune infiltration of HNSCC.

**Fig. 4:**
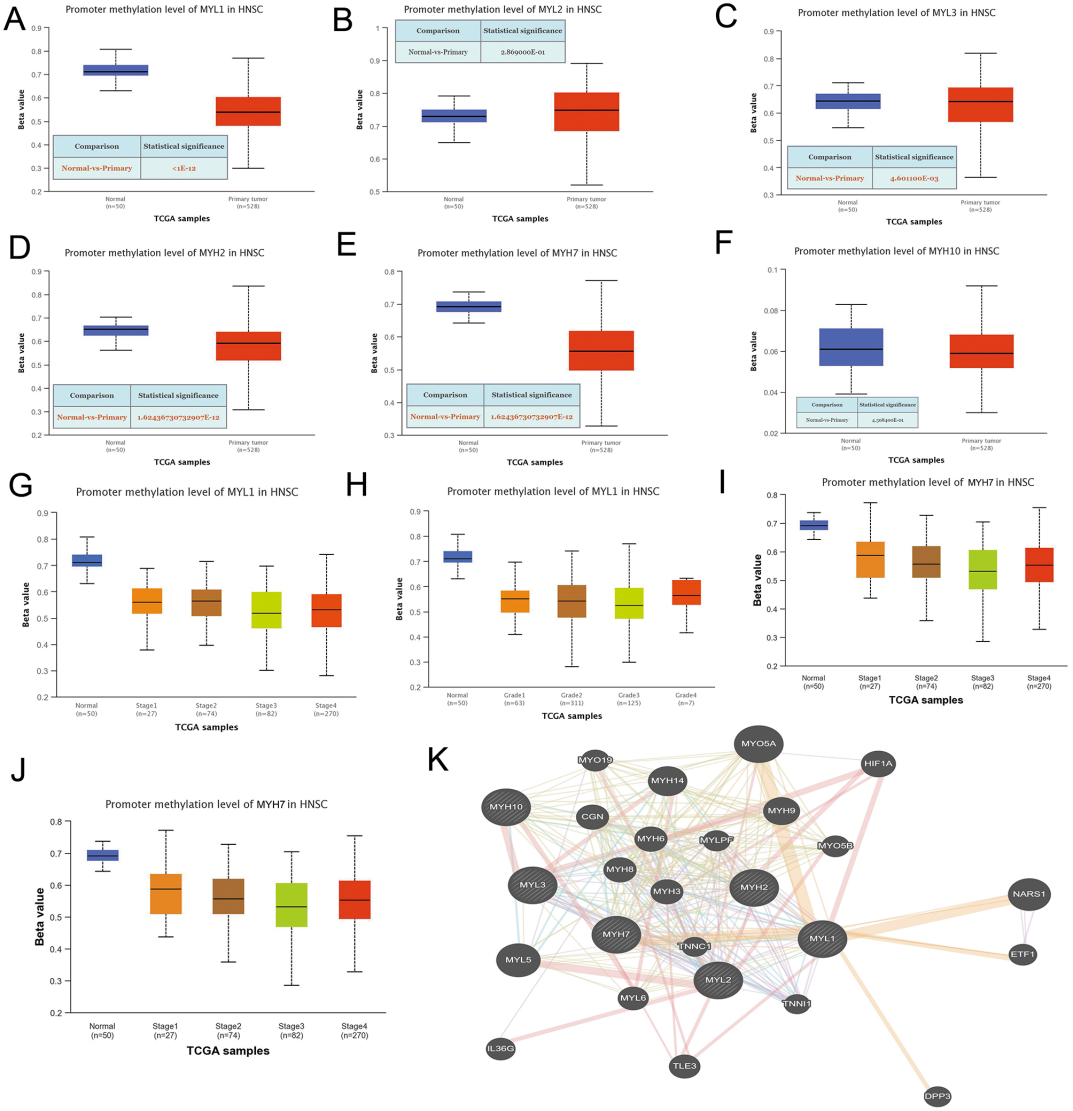
Analysis of methylation level of dysregulated myosin genes and their relationship with tumor stage or grade. **A** Relative methylation level of MYL1 in HNSCC tissues and normal tissues in TCGA HNSCC database. **B** Relative methylation level of MYL2 in HNSCC tissues and normal tissues in TCGA HNSCC database. **C** Relative methylation level of MYL3 in HNSCC tissues and normal tissues in TCGA HNSCC database. **D** Relative methylation level of MYH2 in HNSCC tissues and normal tissues in TCGA HNSCC database **E** Relative methylation level of MYH7 in HNSCC tissues and normal tissues in TCGA HNSCC database. **F** Relative methylation level of MYH10 in HNSCC tissues and normal tissues in TCGA HNSCC database. **G** Correlation analysis of methylation level of MYL1 and tumor stage in TCGA HNSCC database. **H** Correlation analysis of methylation level of MYL1 and tumor grade in TCGA HNSCC database. **I** Correlation analysis of methylation level of MYH7 and tumor stage in TCGA HNSCC database. **J** Correlation analysis of methylation level of MYH7 and tumor grade in TCGA HNSCC database. **K** Possible interactions between dys-regulated MYL and MYH family genes were analyzed by using GeneMANIA.

DNA methylation is closely related to the occurrence and development of cancer. DNA methylation changes include two states: hyper-methylation and hypo-methylation. In general, high methylation level of DNA in the gene promoter region means silencing of the gene, while low methylation level means activation of the gene [23]. We studied methylation level of dys-regulated myosin genes in HNSCC tissues and normal tissues by using UALCAN. The methylation levels of MYL1, MYH2 and MYH7 were significantly down-regulated (P<0.01) (Fig. 4A, D, E), while the methylation level of MYL2 (P>0.01), MYL3 (normal median=0.643, tumor median=0.642, P<0.01) and MYH10 (P>0.01) showed no significant changes in HNSCC tissues compared with normal tissues (Fig. 4B, C, F). There is a statistical significance of MYL1 or MYH7 promoter methylation level between normal tissues and tumor stage 1–4 or grade 1–4 (Fig. 4G-J, S3A-D). Moreover, there is statistical significance of MYL1 expression level between stage 1 and stage 3 or stage 4 (Fig. 4G, S3A). There is statistical significance of MYH7 expression level between stage 1 and stage 3, as well as grade 2 and grade 3 (Fig. 4I, J, S3C, D). Possible interactions between dys-regulated MYL and MYH family genes were analyzed by using Gene MANIA (Fig. 4K).

### MYL1 was down-regulated in HNSCC and promotes HNSCC cells metastasis

**Fig. 5:**
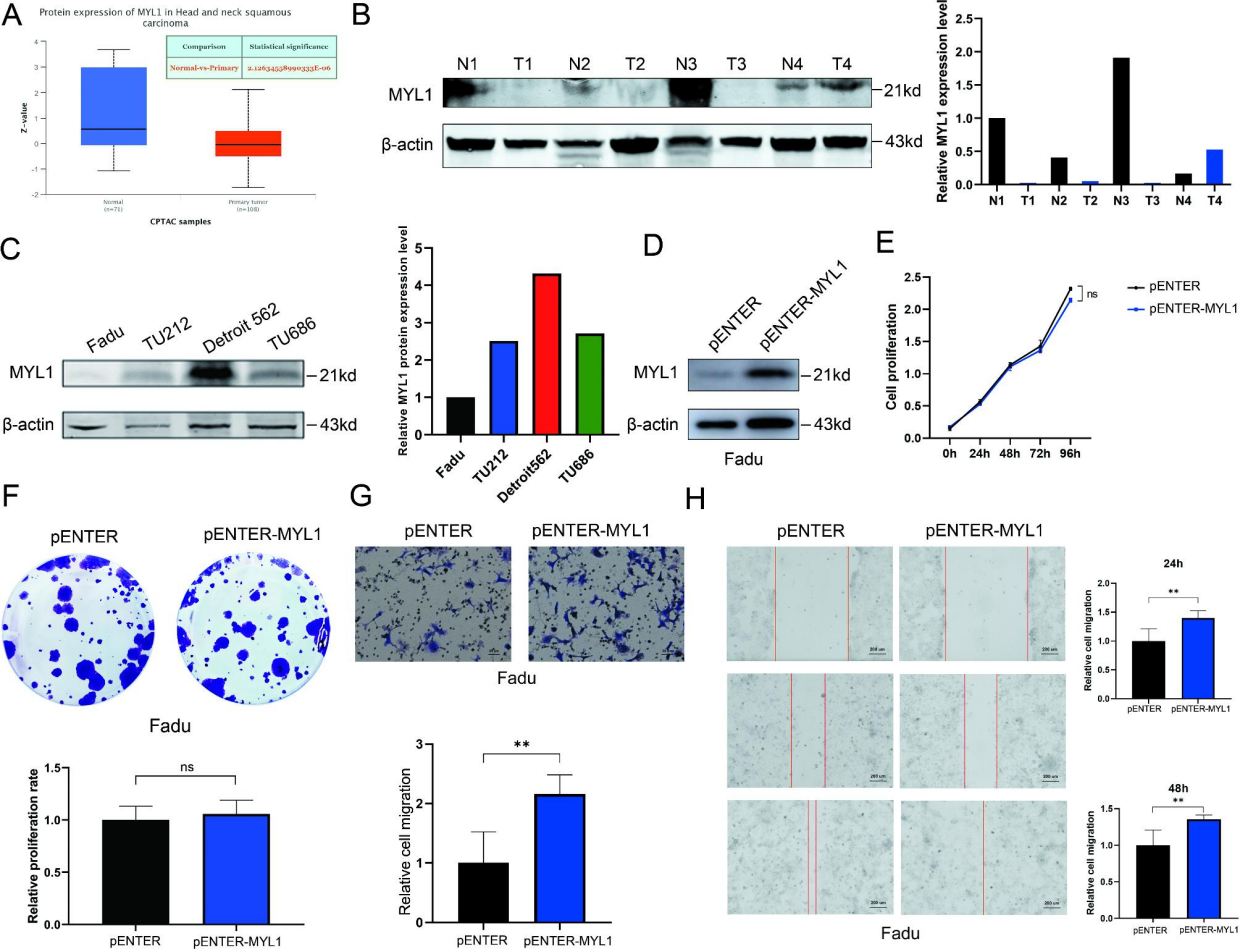
MYL1 was down-regulated in HSCC tissues and promoted Fadu cells metastasis. **A** MYL1 protein expression levels in HSCC tissues and normal tissues were detected by using UALCAN proteomics analysis. **B** MYL1 protein expression levels in HSCC tissues and adjacent normal tissues were detected by using western blot. **C** MYL1 protein expression levels in HNSCC cells. **D** Detection of transfected MYL1 and Control Fadu cells by using western blot. **E** CCK8 assay was used for cell proliferation. (ordinate: OD value. n = 3) **F** Colon assay was used for cell proliferation. (n = 3) **G** Transwell migration assay was used for measuring cell migration capacity at 48 h. (Scale = 50 μm, n = 3,*: P < 0.05;**: P < 0.01) **H** Wound healing assay was used for measuring cell migration capacity at 0, 24 and 48h. (Scale = 200 μm, n = 3,*: P < 0.05;**: P < 0.01)

Since MYL1 showed specifically low expression and methylation level in HNSCC and proved as an unfavorable prognostic marker in HNSCC, we focused on MYL1 for further study. MYL1 protein expression level was down-regulated in HNSCC tissues compared with normal tissues by using UALCAN proteomics analysis (Fig. 5A). We detected MYL1 protein expression levels in tumor and adjacent normal tissue from HSCC patients at stage III or stage IV by using western blot. The results showed MYL1 protein was down-regulated in HSCC (Fig. 5B). We then studied the effects of MYL1 on HNSCC cells. We detected the expression of MYL1 in four HNSCC cell lines by using western blot. MYL1 was relatively highly expressed in Detroit562 cells and lowly expressed in Fadu cells (Fig. 5C). To investigate the function of MYL1 in HNSCC, we constructed MYL1 overexpressed Fadu cell lines and respective negative control cell lines. MYL1 overexpressed cell line was verified by western blot (Fig. 5D). We used CCK8 assay (Fig. 5E) and clone formation assay (Fig. 5F) to study the effect of MYL1 on proliferation of HNSCC cells. The results showed that MYL1 did not affect tumor proliferation. We also used transwell migration assay (Fig. 5G) and wound healing assay (Fig. 5H) to study the effect of MYL1 on metastasis of HNSCC cells. The results showed that MYL1 could promote tumor metastasis. These results together showed that MYL1 could promote metastasis but has little effect on proliferation of HNSCC cells.

### Analysis and verification of MYL1 regulating signaling pathways

**Fig. 6:**
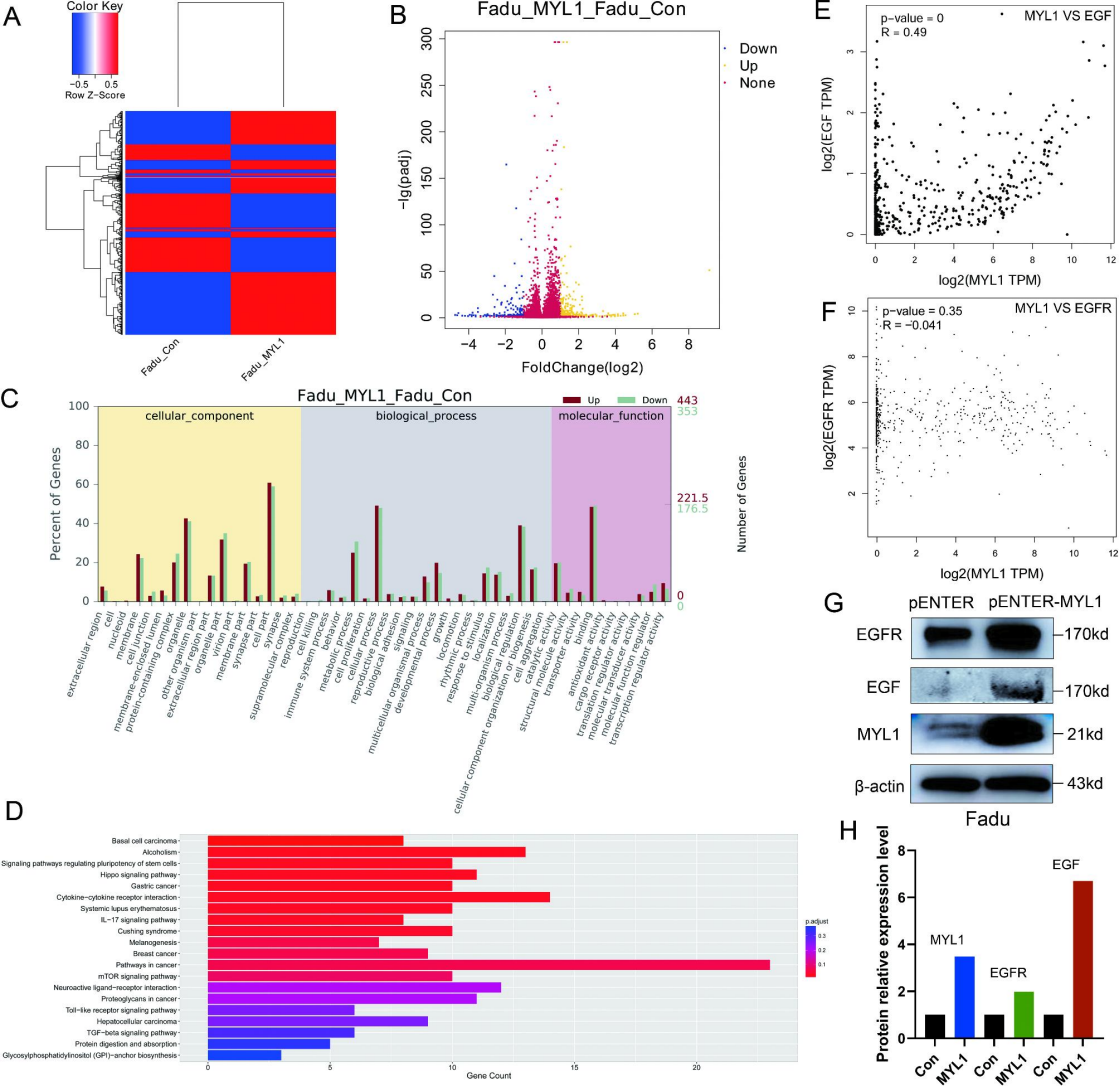
Analysis and verification of MYL1 regulating signaling pathway. **A** The hierarchical cluster analysis diagram revealed different genes between MYL1 overexpressed group and control group. **B** In the volcano diagram, the blue, orange and brown dots represent the down-regulated, up-regulated and no statistically significant differences genes between MYL1 overexpressed group and control group. (FC ≥ 2.0, P < 0.05) **C** Statistical histogram of upregulation and downregulation of differentially expressed genes between MYL1 overexpressed group and control group corresponding to GO function. **D** Statistical histogram of up-regulation and downregulation of differentially expressed genes between MYL1 overexpressed group and control group corresponding to KEGG pathways. **E** Correlation analysis of MYL1 and EGF mRNA in TCGA HNSCC database. **F** Correlation analysis of MYL1 and EGFR mRNA in TCGA HNSCC database. **G** EGF and EGFR protein expression levels in Fadu-MYL1 and Fadu-Con cells were detected by using western blot. **H** Relative expression levels of EGF and EGFR proteins in Fadu-MYL1 and Fadu-Con cells

We then studied the mechanisms of MYL1 promoting HNSCC cells metastasis. The transcriptome sequencing and expression profiling of control Fadu cell lines and MYL1 overexpressing Fadu cell lines were performed to investigate the possible gene signaling pathways regulated by MYL1. Hierarchical cluster analysis and volcano plots showed differences in gene expression between the control group and the MYL1 overexpression group (Fig. 6A, B). Compared with the control group, the expression levels of tumor metastasis-related genes EGF and EGFR were significantly increased in the MYL1 overexpression group. The biological function of differential gene regulation was analyzed by GO functional enrichment prediction (Fig. 6C, D). KEGG analysis showed MYL1 may play a role in promoting tumorigenesis via EGF/EGFR pathway in HNSCC (Figure S4). Gene correlation analysis in TCGA HNSCC database showed there is a positive correlation between MYL1 and EGF at gene level in HNSCC (Fig. 6E), there is no correlation between MYL1 and EGFR at gene level in HNSCC (Fig. 6F). EGF and EGFR increase tumor cell viability, migration and invasion by PI3K/AKT and MEK/ERK pathway [24]. We then studied the effects of MYL1 on EGF and EGFR at protein level by using western blot. The results showed EGF and EGFR protein expression levels were significantly up-regulated in MYL1 over-expressed group compared with control group (Fig. 6G, H). Moreover, there is a positive relationship between EGF, EGFR expression levels and infiltration level of CD4 + T cells in HNSCC (Figure S5A, B). These results indicate MYL1 may promote HNSCC metastasis and CD4 + T cells immune response via EGF/EGFR pathway.

### Correlation between MYL1 and tumor-infiltrating lymphocytes (TILs) in HNSCC

**Fig. 7:**
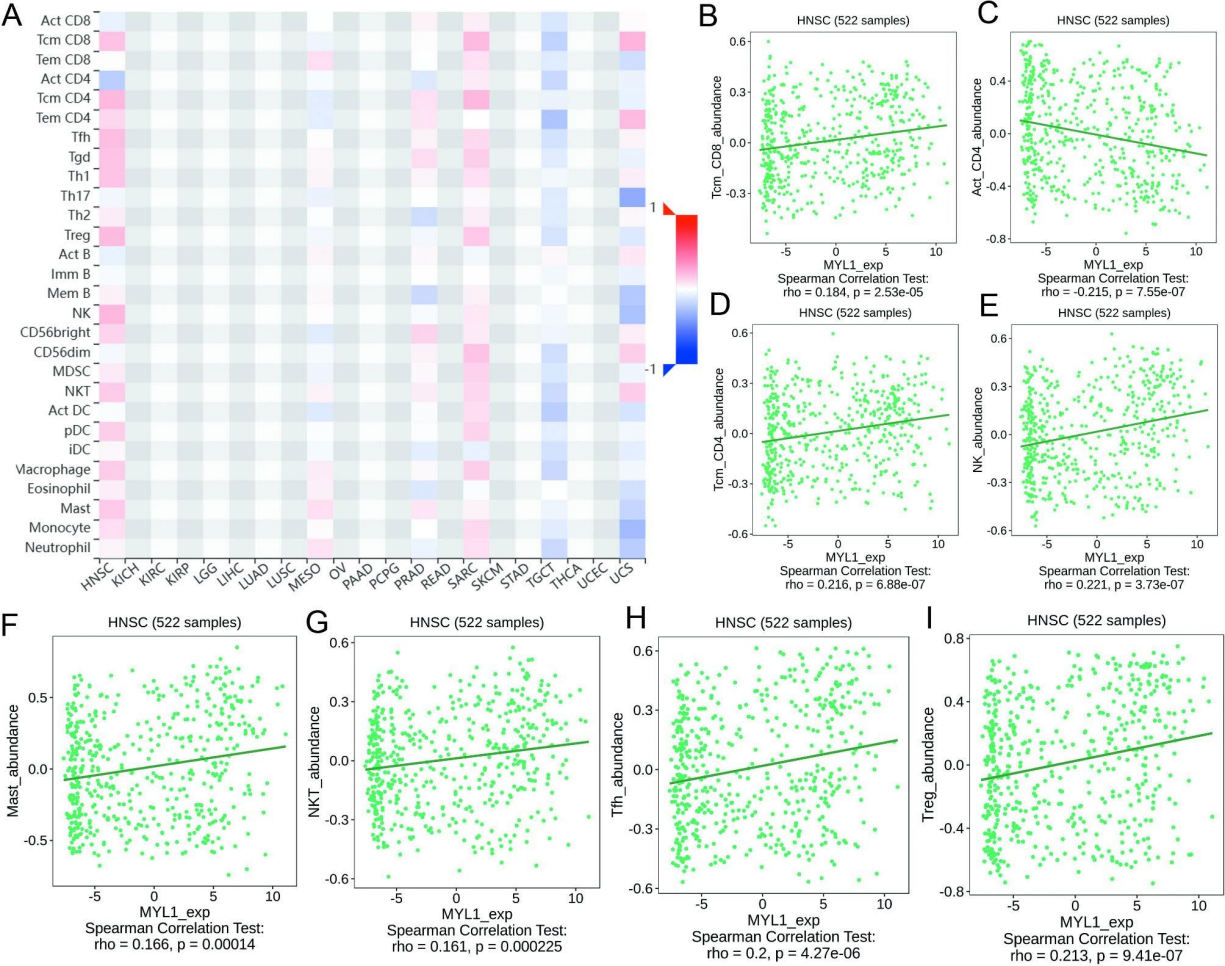
Correlation of MYL1 with tumor-infiltrating lymphocytes (TILs) in HNSCC. **A** Spearman correlations between expression of MYL1 and TILs across human cancers. **B** Spearman correlations between expression of MYL1 and Tcm CD8 cells in HNSCC. **C** Spearman correlations between expression of MYL1 and Act CD4 cells in HNSCC. **D** Spearman correlations between expression of MYL1 and Tcm CD4 cells in HNSCC. **E** Spearman correlations between expression of MYL1 and NK cells in HNSCC. **F** Spearman correlations between expression of MYL1 and Mast cells in HNSCC. **G** Spearman correlations between expression of MYL1 and NKT cells in HNSCC. **H** Spearman correlations between expression of MYL1 and Tfh cells in HNSCC. **I** Spearman correlations between expression of MYL1 and Treg cells in HNSCC

Finally, we studied the correlation between MYL1 expression level and tumor-infiltrating lymphocytes (TILs) in HNSCC in more detail by using TISIDB (Fig. 7A). There is a positive correlation between MYL1 expression and Tcm CD8 cells, Tcm CD4 + cells, NK cells, Mast cells, NKT cells, Tfh cells or Treg cells (r>0.1, P<0.01) (Fig. 7B, D-I), there is only a negative correlation between MYL1 expression and Act CD4 cells (r<-0.1, P<0.01) (Fig. 7C). These results indicate MYL1 promotes immune response in HNSCC.

## Discussion

Most of head and neck cancers are squamous-cell cancers, which are related to human papilloma virus infection, tobacco and alcohol [25]. In recent years, the mortality rate of patients with squamous cell carcinoma of the head and neck has been high, which poses a serious threat to human health [26]. Metastasis and the associated loss of function of vital organs in the course of the disease is one of the main causes of death in head and neck cancer patients [27]. Although factors such as pathological grade, tumor stage, and lymph node metastasis are related to the prognosis of HNSCC patients, accurate evaluation marker of prognosis in HNSCC patients is very lacking. ​Therefore, it is important to search for novel prognostic biomarkers and molecular targets that can be used for the diagnosis and treatment of HNSCC.

In recent years, bioinformatics analysis has been widely used in predicting biomarkers for diagnosis and treatment in various cancers. High throughput genome and bioinformatics analysis have become an effective means to find potential targets for the diagnosis and treatment of tumors. The Cancer Genome Atlas (TCGA) is the world’s largest cancer genetic information database. It is a multi-omics database, including the genome, transcriptome, proteome, epigenetic data of common cancers and related clinical data. NAT10 [28], CCT3 [29], PLEK2 [30], HOXB7 [31], FOXD1 [32], SEC61G [33], and so on have been identified as tumor markers in HNSCC by performing TCGA HNSCC database analysis.

Our research specifically analyzed myosin genes in HNSCC. Myosin genes MYL1, MYL2, MYH2, and MYH7 are down-regulated in HNSCC. Interestingly, relative high expression of these genes is related to poor prognosis of HNSCC. MYL1, MYL2, MYH2, and MYH7 expression levels were significantly down-regulated in stage II, III, IV compared with stage I, MYL1 is a potential special poor prognostic marker in HNSCC. Xu et al. found MYL1, MYH6 and MYH8 down-regulated and MYL1 as a poor prognostic gene in HNSCC [34], which are consistent with our study. In the conventional sense, high expression of down-regulated genes in tumors is always associated with a good prognosis for the tumor, our findings break from that routine. There is a positive relationship between the expression levels of MYL1, MYL2, MYL3, MYH2, MYH7, MYH10 and infiltration level of CD4 + T cells. These results provide novel diagnosis and treatment targets for HNSCC.

Our further findings suggest that MYL1 is down-regulated in HSCC tissues compared with normal tissues at protein levels. MYL1 overexpression has no effect on proliferation, but significantly promotes migration of HSCC cells. MYL1 enhances protein expression levels of EGF and EGFR in HSCC. There is a positive correlation between MYL1 expression and Tcm CD8 cells, Tcm CD4+ cells, NK cells, Mast cells, NKT cells, macrophages cells or Treg cells in HNSCC. Depending on the different roles of immune cells in the microenvironment, tumor-infiltrating immune cells can be divided into tumor-suppressing and tumor-promoting immune cells. NK cells, M1 cells, N1 cells, dendritic cell 1 (DC1 cells), T helper cell 1 (Th1 cells), and CD8+T cells are tumor suppressive immune cells. MDSC cells, M2 cells, N2 cells, DC2 cells, Th2 cells and regulatory T cells (Treg cells) belong to tumor promoting immune cells. The cytokines produced by these cells play a central role in shaping the tumor microenvironment, thereby driving immune-mediated anti-tumor or pro-tumor activity in the tumor microenvironment [35]. Overall, MYL1 promotes metastasis and correlates with tumor immune infiltration in HNSCC, which may explain why MYL1 is down-regulated but its relative high expression level is associated with a poor prognosis in HNSCC, and provides new insights into the mechanisms of HNSCC.  These effects of MYL1 may be related to the EGF/EGFR signaling pathway. However, the underlying mechanism of MYL1 promoting tumor needs to be further verified.

It may seem paradoxical but true that the expression of myosin genes in patients with HNSCC are significantly down-regulated, but their relatively high expression is a marker of poor prognosis for HNSCC. We suppose in the process of tumorigenesis, certain traits of muscle cells such as pharyngeal muscle change and lose their functions, leading to down–regulated of myosin genes. On the other hand, myosin genes may play roles in promoting tumor development and metastasis, which induce short overall survival time of patients with relative high expression of myofibrillar genes.

## Conclusion

We conclude that myosin genes MYL1, MYL2, MYH2, and MYH7 are significantly down-regulated but proved as unfavorable prognostic markers in HNSCC. MYL1 facilitates tumor metastasis and correlates with tumor immune infiltration in HNSCC and these effects may be associated with the EGF/EGFR pathway.

### Electronic supplementary material

Below is the link to the electronic supplementary material.

Supplementary Material 1

Supplementary Material 2

Supplementary Material 3

Supplementary Material 4

Supplementary Material 5

Supplementary Material 6

## Data Availability

The datasets analyzed during the current study are available in the GEO repository, accession no. GSE58911, GSE30784.The data that support the findings of this study are available from the corresponding author upon reasonable request.

## Abbreviations

HNSCC: Head and neck squamous cell carcinoma
HSCC: Hypopharyngeal squamous cell carcinoma
OSCC: Oral squamous cell carcinoma
SD: Standard deviation
TCGA: The Cancer Genome Atlas
TILs: Tumor-infiltrating lymphocytes
