# Supplementary material for: Analysis of myosin genes in HNSCC and identify MYL1 as a specific poor prognostic biomarker, promotes tumor metastasis and correlates with tumor immune infiltration in HNSCC
Source: BMC Cancer. 2023 Sep 7;23:840. doi: 10.1186/s12885-023-11349-5 (PMC10486092; doi:10.1186/s12885-023-11349-5)
Supplement: Supplementary file 1 — Supplementary Material 1 [file 12885_2023_11349_MOESM1_ESM.pdf]

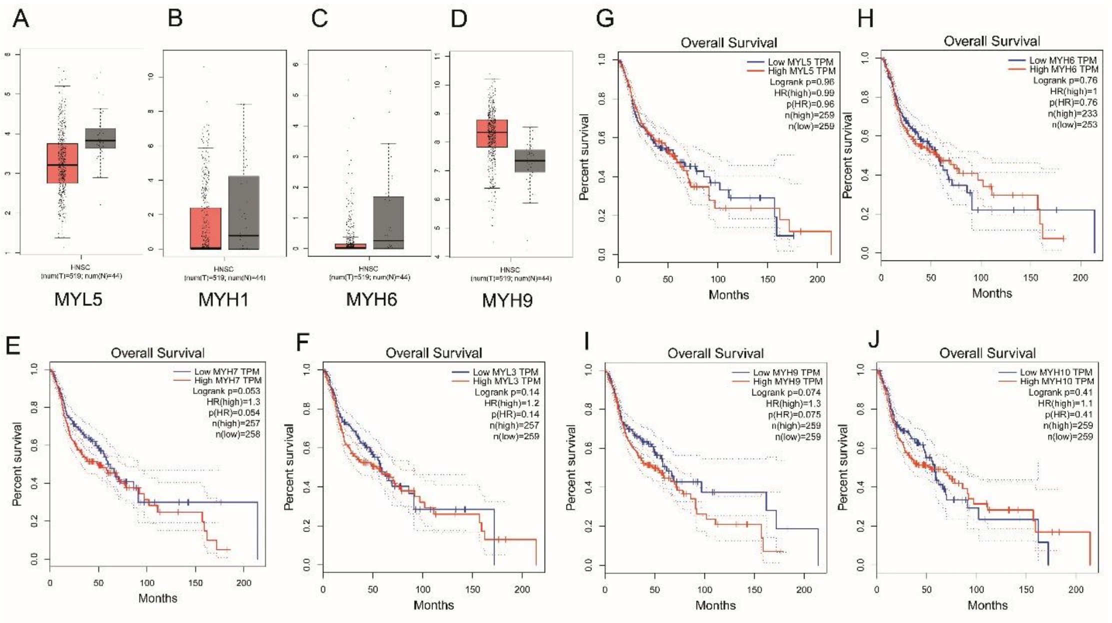

Figure S1 Gene chip analysis of myosin genes in head and neck squamous cell carcinoma and expression level and survival time analysis in TCGA database.

A MYL5 expression level (log_2(TPM+1)) in HNSCC patients and normal persons in TCGA HNSCC database. **B** MYH1 expression level (log_2(TPM+1)) in HNSCC patients and normal persons in TCGA HNSCC database. **C** MYH6 expression level (log_2(TPM+1)) in HNSCC patients and normal persons in TCGA HNSCC database. **D** MYH9 expression level (log_2(TPM+1)) in HNSCC patients and normal persons in TCGA HNSCC database. **E** Correlation analysis of survival and MYH7 expression in HNSCC patients in TCGA HNSCC database. **F** Correlation analysis of survival and MYL3 expression in HNSCC patients in TCGA HNSCC database. **G** Correlation analysis of survival and MYL5 expression in HNSCC patients in TCGA HNSCC database. **H** Correlation analysis of survival and MYH6 expression in HNSCC patients in TCGA HNSCC

database. **I** Correlation analysis of survival and MYH9 expression in HNSCC patients in TCGA HNSCC database. **J** Correlation analysis of survival and MYH10 expression in HNSCC patients in TCGA HNSCC database.
