# Supplementary material for: Analysis of myosin genes in HNSCC and identify MYL1 as a specific poor prognostic biomarker, promotes tumor metastasis and correlates with tumor immune infiltration in HNSCC
Source: BMC Cancer. 2023 Sep 7;23:840. doi: 10.1186/s12885-023-11349-5 (PMC10486092; doi:10.1186/s12885-023-11349-5)
Supplement: Supplementary file 2 — Supplementary Material 2 [file 12885_2023_11349_MOESM2_ESM.pdf]

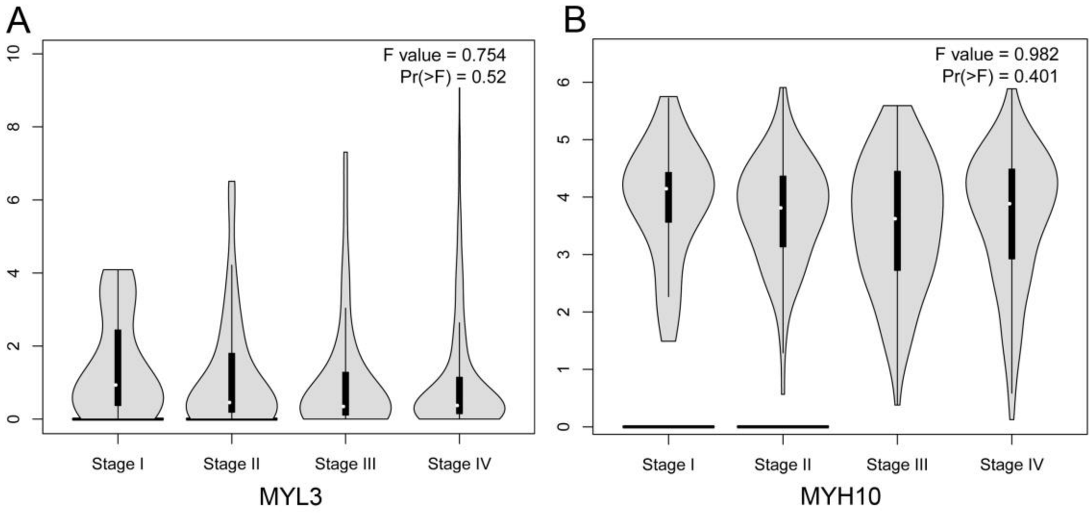

Figure S2 Correlation analysis between tumor stage and expression levels of dysregulated myosin genes.

A Correlation analysis of MYL3 and tumor stage in TCGA HNSCC database. **B** Correlation analysis of MYH10 and tumor stage in TCGA HNSCC database.
