# Supplementary material for: Analysis of myosin genes in HNSCC and identify MYL1 as a specific poor prognostic biomarker, promotes tumor metastasis and correlates with tumor immune infiltration in HNSCC
Source: BMC Cancer. 2023 Sep 7;23:840. doi: 10.1186/s12885-023-11349-5 (PMC10486092; doi:10.1186/s12885-023-11349-5)
Supplement: Supplementary file 3 — Supplementary Material 3 [file 12885_2023_11349_MOESM3_ESM.pdf]

A Promoter methylation level of MYL1 in HNSC

Comparison	Statistical significance
Normal-vs-Stage1	3.22297744048683E-13
Normal-vs-Stage2	<1E-12
Normal-vs-Stage3	1.62436730732907E-12
Normal-vs-Stage4	<1E-12
Stage1-vs-Stage2	2.926800E-01
Stage1-vs-Stage3	4.914300E-02
Stage1-vs-Stage4	3.439600E-02
Stage2-vs-Stage3	1.802750E-01
Stage2-vs-Stage4	1.097170E-01
Stage3-vs-Stage4	9.735000E-01

B Promoter methylation level of MYL1 in HNSC

Comparison	Statistical significance
Normal-vs-Grade1	<1E-12
Normal-vs-Grade2	1.62447832963153E-12
Normal-vs-Grade3	1.62447832963153E-12
Normal-vs-Grade4	1.82200032838864E-10
Grade1-vs-Grade2	5.713000E-01
Grade1-vs-Grade3	5.994200E-01
Grade1-vs-Grade4	4.975200E-01
Grade2-vs-Grade3	9.327600E-01
Grade2-vs-Grade4	3.922600E-01
Grade3-vs-Grade4	4.408400E-01

C Promoter methylation level of MYH7 in HNSC

Comparison	Statistical significance
Normal-vs-Stage1	2.04410000037569E-07
Normal-vs-Stage2	1.62447832963153E-12
Normal-vs-Stage3	<1E-12
Normal-vs-Stage4	<1E-12
Stage1-vs-Stage2	2.014600E-01
Stage1-vs-Stage3	1.667790E-02
Stage1-vs-Stage4	5.225900E-02
Stage2-vs-Stage3	5.778400E-02
Stage2-vs-Stage4	3.439200E-01
Stage3-vs-Stage4	1.410970E-01

D Promoter methylation level of MYH7 in HNSC

Comparison	Statistical significance
Normal-vs-Grade1	1.62447832963153E-12
Normal-vs-Grade2	1.62447832963153E-12
Normal-vs-Grade3	1.62447832963153E-12
Normal-vs-Grade4	5.963700E-04
Grade1-vs-Grade2	5.145800E-01
Grade1-vs-Grade3	2.602200E-01
Grade1-vs-Grade4	2.446600E-01
Grade2-vs-Grade3	1.794250E-02
Grade2-vs-Grade4	2.116200E-01
Grade3-vs-Grade4	5.292800E-01

Figure S3 Correlations between promoter methylation level of MYL1 or MYH7 and individual tumor stages or tumor grades in HNSCC.

A Correlations between promoter methylation level of MYL1 and individual tumor stages in HNSCC. **B** Correlations between promoter methylation level of MYL1 and tumor grades in HNSCC. **C** Correlations between promoter methylation level of MYH7 and individual tumor stages

in HNSCC. **D** Correlations between promoter methylation level of MYH7 and tumor grades in HNSCC.
