# Supplementary figures and images for: Analysis of myosin genes in HNSCC and identify MYL1 as a specific poor prognostic biomarker, promotes tumor metastasis and correlates with tumor immune infiltration in HNSCC
Source: BMC Cancer. 2023 Sep 7;23:840. doi: 10.1186/s12885-023-11349-5 (PMC10486092; doi:10.1186/s12885-023-11349-5)

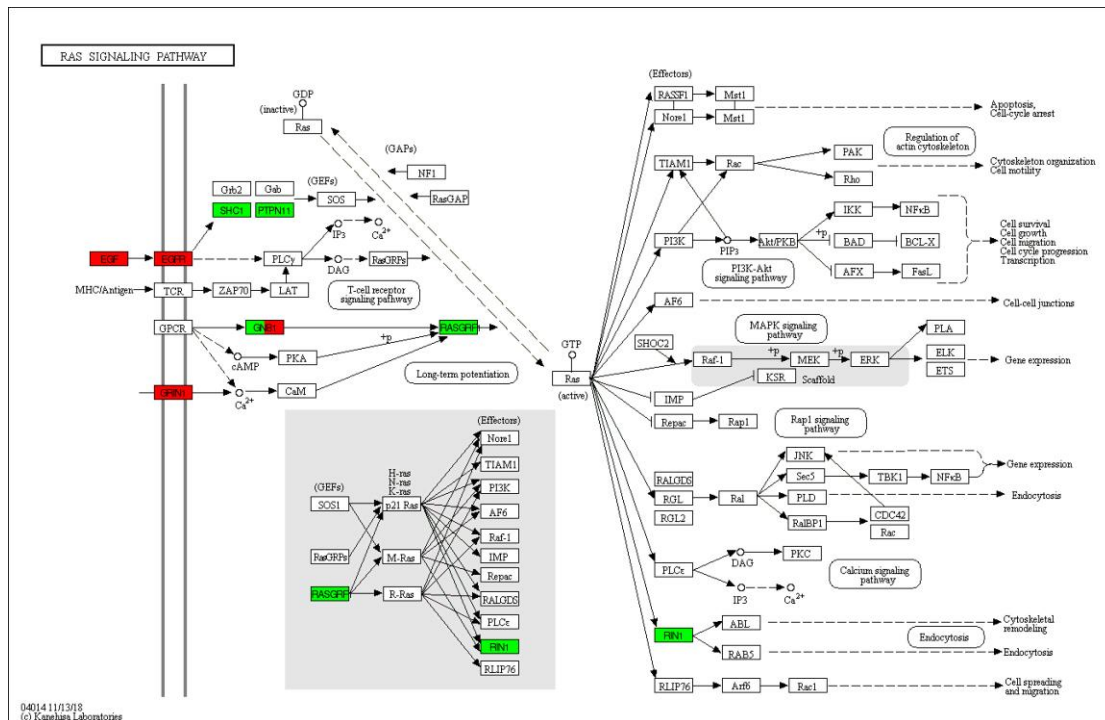

**Figure S4 The effects of MYL1 on RAS signaling pathway**

Supplement: Supplementary file 4 — Supplementary Material 4 [file 12885_2023_11349_MOESM4_ESM.pdf]

Original Western blot strip

Figure 5A

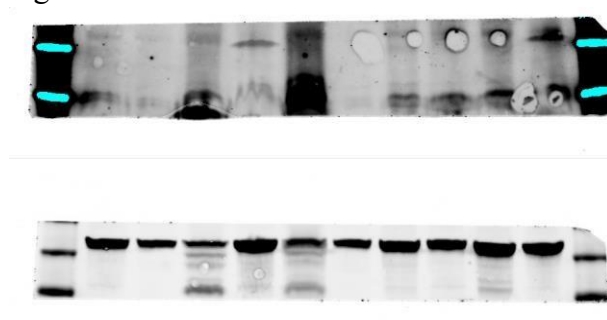

Figure 5C

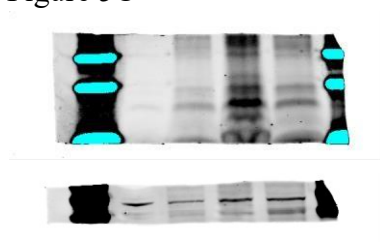

Figure 5D

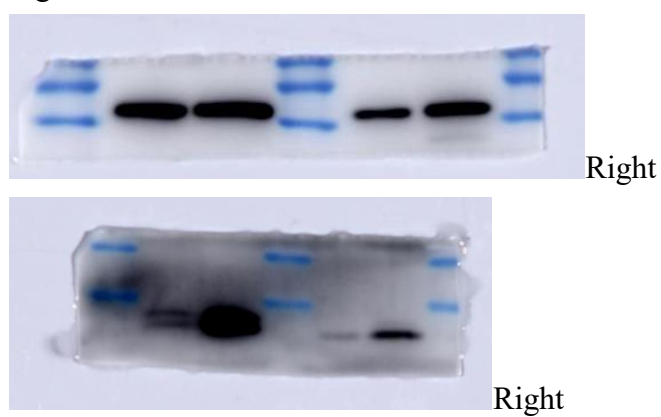

Figure 6G

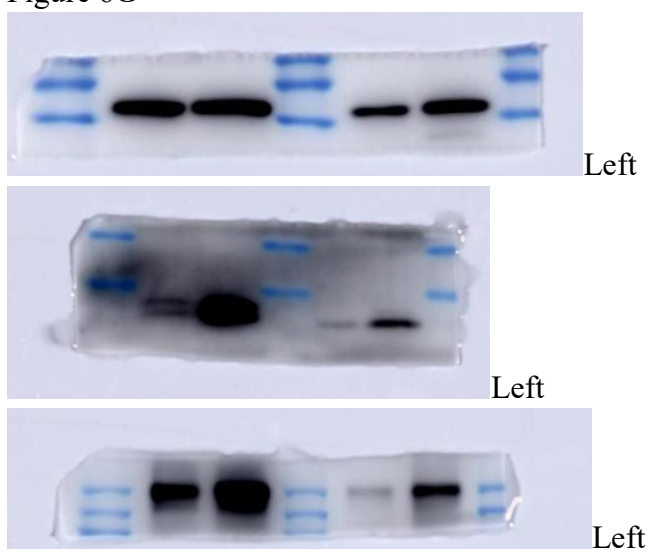

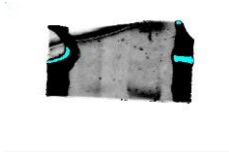

Supplement: Supplementary file 6 — Supplementary Material 6 [file 12885_2023_11349_MOESM6_ESM.pdf]
