# Supplementary material for: Analysis of myosin genes in HNSCC and identify MYL1 as a specific poor prognostic biomarker, promotes tumor metastasis and correlates with tumor immune infiltration in HNSCC
Source: BMC Cancer. 2023 Sep 7;23:840. doi: 10.1186/s12885-023-11349-5 (PMC10486092; doi:10.1186/s12885-023-11349-5)
Supplement: Supplementary file 5 — Supplementary Material 5 [file 12885_2023_11349_MOESM5_ESM.pdf]

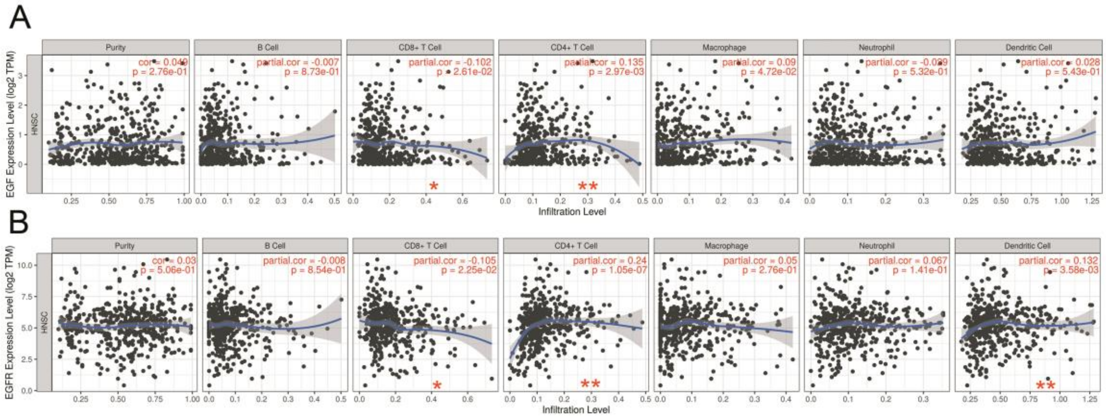

Figure S5 Immune infiltration of EGF and EGFR in HNSCC.

A Immune infiltration of EGF in HNSCC. **B** Immune infiltration of EGFR in HNSCC. (*: P<0.05, **: p<0.01)
